# “[T]he laws need to change to reflect current society”: Insights from stakeholders involved in development, review or implementation of policies about adolescent consent for HIV testing, care and research in Kenya

**DOI:** 10.1002/jia2.26057

**Published:** 2023-01-15

**Authors:** Huangqianyu Li, Seema K. Shah, Elise Healy, Kawango Agot, Jillian Neary, Kate Wilson, Jacinta Badia, Winnie O. Atieno, Hellen Moraa, Hendrika Meischke, James Kibugi, Irene Inwani, Nok Chhun, Ferdinand C. Mukumbang, Grace John‐Stewart, Pamela Kohler, Kristin Beima‐Sofie

**Affiliations:** ^1^ Department of Health Services University of Washington Seattle Washington USA; ^2^ Department of Pediatrics Northwestern University Feinberg School of Medicine Chicago Illinois USA; ^3^ Bioethics Program at Lurie Children's Hospital Chicago Illinois USA; ^4^ Department of Global Health University of Washington Seattle Washington USA; ^5^ Impact Research and Development Organization Kisumu Kenya; ^6^ Department of Medicine University of Washington Seattle Washington USA; ^7^ Department of Pediatrics and Child Health University of Nairobi Nairobi Kenya; ^8^ University of Nairobi/Kenyatta National Hospital Nairobi Kenya; ^9^ Department of Epidemiology University of Washington Seattle Washington USA; ^10^ Department of Pediatrics University of Washington Seattle Washington USA; ^11^ Department of Child Family and Population Health Nursing University of Washington Seattle Washington USA

**Keywords:** adolescent HIV, HIV/AIDS research, bioethics, consent, decision‐making, research regulation

## Abstract

**Introduction:**

Engaging adolescents in HIV care and research promotes the development of interventions tailored to their unique needs. Guidelines generally require parental permission for adolescents to receive HIV care/testing or participate in research, with exceptions. Nevertheless, parental permission requirements can restrict adolescent involvement in care and research. To better appreciate prospects for policy reform, we sought to understand the perspectives of stakeholders involved in the development, review and implementation of policies related to adolescents living with HIV.

**Methods:**

Semi‐structured individual interviews (IDIs) were conducted from October 2019 to March 2020 with 18 stakeholders with expertise in the (1) development of policy through membership in the Law Society of Kenya or work as a health policy official; (2) review of policy through ethics review committee service; or (3) implementation of policy through involvement in adolescent education. IDIs were conducted in English by Kenyan social scientists, audio‐recorded and transcribed verbatim. We used thematic analysis to identify themes around how policies can be reformed to improve adolescent engagement in HIV care and research.

**Results:**

Our analysis identified three major themes. First, policies should be flexible rather than setting an age of consent. Stakeholders noted that adolescents’ capacity for engagement in HIV care and research depended on context, perceived risks and benefits, and “maturity”—and that age was a poor proxy for the ability to understand. Second, policies should evolve with changing societal views about adolescent autonomy. Participants recognized a generational shift in how adolescents learn and mature, suggesting the need for a more frequent review of HIV care and research guidelines. Third, adults should empower adolescent decision‐making. Stakeholders felt that caregivers can gradually involve adolescents in decision‐making to equip them to gain ownership over their health and lives, improving their confidence and capacity.

**Conclusions:**

Revising relevant laws to consider context, alternative measures of maturity, and evolving societal views about adolescence, along with supporting caregivers to assist in developing adolescent autonomy may promote more equitable and representative participation of adolescents in HIV care and research. Additional research should explore how to support caregivers and other adults to empower adolescents and improve stakeholder engagement in a more routine process of policy reform.

## 1 INTRODUCTION

Growing numbers of adolescents (ages 10–19) are living with HIV (ALHIV) [1]. There is an urgent need for representative participation of ALHIV in HIV testing, care and research on interventions tailored to this population. Compared with adults, adolescents have distinct physiological, psychological, and developmental needs and health behaviours [2], along with lower adherence to therapy and increased mortality from HIV [3, 4]. ALHIV care linkage and retention remain poorly understood [5].

Despite the importance of including adolescents in HIV testing, care and research [6], consent requirements for adolescents are confusing and variable, impeding adolescent inclusion [7]. For example, in Kenya, consent processes generally require parental or caregiver permission until adolescents are 18, but with many exceptions based on the adolescent's illness, status (e.g. marital status or military involvement) and maturity, and whether consent is for testing, care or research. Caregivers are adults who are recognized, either legally or by the community, as authorized to make decisions for particular adolescents [8]. For HIV testing, adolescents 15 and older can independently consent, but must identify a primary caregiver when enrolling in HIV care and initiating antiretroviral therapy (ART) [9, 10]. Adolescents younger than 15 may independently consent for testing if they are “symptomatic [for HIV/AIDS], pregnant, married, a parent, or engaged in behavior that puts them at risk of contracting HIV [11].” For research, parental permission is required unless adolescents are “mature” or emancipated minors; the IRB grants a waiver of parental consent (e.g. for orphans heading households, adolescents who are pregnant or parents and those living in institutions); or the IRB determines that parental permission is not a reasonable protection (e.g. research on child abuse). Guidelines also permit adolescents older than 12 to consent independently for research on sexually transmitted diseases [8]. How these guidelines and exceptions are understood and implemented, however, is unclear. Despite calls for legal reform, such policies can be hard to change [12, 13].

When adolescents are not permitted to consent independently, caregivers must be engaged in decision‐making. While this works well for some adolescents [14, 15, 16], those with caregivers who are unavailable or unwilling to consent to treatment could lose access to potentially life‐saving benefits. In research, requiring caregiver involvement for adolescents concerned about disclosing their sexual behavior and/or HIV status can make it difficult to enrol groups with adherence challenges, who are especially important to study [5, 17, 18].

Parental or caregiver permission is typically seen as a way to protect adolescents with limited decision‐making capacity and the ability to protect their interests [19, 20]. Although some adolescents who are wary of parental involvement may have other trusted adults who function as caregivers, these caregivers may not be recognized as having authority to provide consent. Moreover, while evidence suggests that adolescents aged 14 years and older have similar decision‐making capacity to adults [21, 22], there is no definitive age at which most adolescents have the same capacity for understanding as adults. Finally, there is limited research on adolescent decision‐making capacity in low‐ and middle‐income countries [12, 23].

Various groups are responsible for developing, reviewing, interpreting and implementing laws and policies related to adolescent consent. These policy stakeholders have unique insights into how policies are working, if they require reform, and what reform should entail. Within the Data‐informed Stepped Care (DiSC) to Improve Adolescent HIV Outcomes study [24], we conducted qualitative research with stakeholders from a variety of backgrounds (including adolescents, parents, caregivers and healthcare workers) on adolescent engagement in HIV testing, care and research. In this paper, we present qualitative analysis focusing on the perspectives of stakeholders with experience implementing, reviewing and designing relevant policies. The goal of this study was to identify perspectives on existing policies from those who have relevant experience to identify recommendations for reform.

## 2 METHODS

### 2.1 Study design and population

We conducted a qualitative study, nested within DiSC, a clinical trial in Kenya designed to optimize the provision of clinical care and improve retention among ALHIV in two counties in Western Kenya, Kisumu and Homa Bay [25]. We follow the guidelines for reporting qualitative research below [26].

Within this qualitative sub‐study, we sought to understand views on adolescent decision‐making, autonomy and caregiver support related to HIV testing, care and research, from the perspective of policy stakeholders in Kenya, to identify proposals for and understand the viability of policy reform. Policy stakeholders were defined as those developing policy by working as attorneys who are part of the Law Society of Kenya or as public health officials, those applying policies through involvement in school administration, or interpreting policy by serving on ethical review committees. Purposive sampling was used to recruit participants representing a broad range of professional expertise. Potential participants were identified through professional networks, public databases and other in‐country resources. Potential participants were contacted by study staff by phone or email, and if unable to participate, asked to suggest additional participants with similar expertise. Interviews were conducted until information power was reached [27], in line with widely referenced sample size estimations for qualitative work using purposive sampling [28, 29].

### 2.2 Data collection

Each participant completed a single semi‐structured interview, conducted between October 2019 and March 2020. The semi‐structured interview guide (Supplementary Material 1) was developed based on a literature review of policy considerations for adolescent engagement in HIV testing, care and research, along with previously published and in‐progress qualitative research with other stakeholder groups. The interview guide probes policy stakeholders’ experiences with and beliefs about laws governing adolescent decision‐making related to: (1) considerations for adolescent decision‐making; (2) adolescent consent to and participation in research; and (3) optimal policy approaches for adolescent decision‐making. The interview guide was piloted with study staff and subsequently revised for optimal phrasing, timing and ordering of questions.

Before each interview, we collected basic demographic information, including age, sex, educational level, occupation and years of experience. Interviews were conducted in English at clinics by two trained Kenyan social scientists with no prior relationships with research participants. All interviews were audio‐recorded and transcribed verbatim. Structured debrief reports were completed by interviewers immediately after each interview [30]. Interviews lasted an average of 40 minutes.

### 2.3 Data analysis

Thematic analysis was performed using a team‐based coding approach consisting of a core team of four coders (KBS, SS, EH and HL) using *ATLAS.ti* software (version 8). The team performed conventional content analysis [31] using the constant comparison method to produce a description of themes identified within and between the individual primary categories represented in the interview guide. An initial structured codebook was developed using open coding, by two coders (HL and EH), after reviewing the full set of debrief reports and a subset of transcripts. Codebook development was guided by published literature on parental permission requirements and adolescent autonomy [12, 32, 33, 34, 35, 36, 37], and a previous codebook was used to analyse qualitative data from other stakeholders (adolescents, caregivers and healthcare workers) participating in additional qualitative research activities within DiSC. The initial codebook was refined by the larger coding team through an iterative process of reviewing additional transcripts against the existing codebook, reviewing code application in team meetings and refining based on team feedback.

All coders participated in a consensus coding process. Following consensus coding, the final version of the codebook was used to code all transcripts. Three coders (KBS, EH and HL) each independently coded a randomly selected subset of transcripts. All coded transcripts were then reviewed by a second member of the coding team (KBS, EH, HL and SS) to assess agreement with text segmentation, code application and interpretation. Disagreements in code application and segmentation were resolved through group discussions until a consensus was reached. The analysis team drafted and revised thematic memos throughout the analysis process. Memos, queries and code co‐occurrence tables were used to identify patterns within and across transcripts and inform the development of the themes related to adolescent autonomy and consent practices [38, 39]. Thematic network analysis techniques were used to refine, characterize and map the network of related themes regarding the main domains investigated [40]. Despite having to halt recruitment due to the COVID pandemic, we believe we reached sufficient information power [27] to capture relevant considerations for policies related to adolescent HIV decision‐making from the perspective of these key stakeholders.

### 2.4 Ethical considerations

This study was reviewed and approved by the Maseno University Ethical Review Committee in Kenya and the University of Washington Institutional Review Board. All participants provided written informed consent.

## 3 RESULTS

A total of 18 policy stakeholders participated in individual interviews. Two recordings were corrupted, and responses from these two interviewees were indirectly obtained from debrief reports prepared by the interviewer rather than from transcripts or direct quotes. Stakeholders included IRB/ERC members (*N* = 3), lawyers (*N* = 4), public health officials (*N* = 7) and school administrators/teachers (*N* = 4) (Table 1). Stakeholders were a median of 40 years of age and the majority (69%) were male. Nine participants (50%) reported ever having an adolescent child of their own. All participants had university‐level education. Most reported being familiar with policies or guidelines on adolescent autonomy (81%) and having previously participated in a research study themselves (88%), prior to this one.

**Table 1:** Demographic characteristics of policy stakeholders

**Characteristic**	***N* (%) or Median (IQR)** **^a^**
** Occupation**	
* IRB/ERC Member*	4 (22)
* Lawyer*	4 (22)
* Policymaker*	7 (39)
School administrator	3 (17)
** Male**	11 (69)
** Median age (IQR)**	40 (35–49)
Has experience as caregiver of an adolescent	10 (63)
** Current caregiver of an adolescent**	9 (56)
** Aware of guidelines/policies on adolescent autonomy**	13 (81)
** Previous participation in research**	14 (88)

^a^Total *N* = 18.

Stakeholders identified three overarching considerations related to policies and guidelines for adolescent decision‐making and consent practices in HIV testing, care and research. These considerations balanced stakeholder's perceptions of adolescents’ cognitive capacity, knowledge of HIV and health, available support systems and current guidelines related to adolescent decision‐making. Stakeholders described how guidelines responsive to multifaceted considerations may improve adolescent engagement in HIV testing, care and research.

### 3.1 Adolescent capacity for independent decision‐making varied based on context, risk/benefit ratio and maturity, but age is a poor proxy

All participants agreed that adolescents were frequently involved in decision‐making about their lives and health, though independent decisions were largely limited to what participants considered lower‐risk circumstances, including daily tasks and responsibilities, and experiences at school (e.g. which classes to take). Most participants believed that adolescents could also make independent decisions related to their health based on the personal nature of health problems, their maturity and the urgency involved.

“[H]ealth issues are going to be very personal, and if you are going to have an adolescent who has already decided on what they want, especially when they are going to utilize a particular health product, which has been researched on and approved either internationally and at the national level and that has been found to be working and has been rolled out…I think at that point…they can be allowed to make their own decision…”—[Participant 8, IRB/ERC Member]

Most participants considered HIV care as distinct from general health services, in terms of knowledge and support needed to understand the care and cope with HIV status and lifelong antiretrovirals. Most stakeholders generally supported independent adolescent decision‐making for HIV care if risks and benefits were carefully discussed. However, participants also suggested caregiver involvement to facilitate future adherence when possible, raising concerns about adolescents not having sufficient support to cope with testing positive for HIV.

“It's advisable to notify the parent or give some background information about what's being done so that maybe the parent or the guardian is aware … at the end of the day, the parent has to know because the parent now will be forced to come in and support nutritionally, in adherence, psychosocial support, clinic days, and in school.”—[Participant 6, Public Health Official]

Because participants felt that participating in research generally involved greater net risk than receiving care, many wanted additional protections before they would feel comfortable permitting independent adolescent consent for research participation. Participants described needing to evaluate the study design, ensuring that an IRB or REC had given approval, and determining that there was a compelling need to enrol adolescents in research. Participants also emphasized ensuring that adolescents were benefiting from participation and not being exploited by researchers.

“It depends on the content of the study; what the study is up to, if it is something that is geared towards the adolescents, then when they become part of that study, then I think they will move forward to gaining from that study.”—[Participant 3, School Teacher/Administrator]

Although policies often determine when adolescents can give independent consent for testing, care and research based on their age, stakeholders noted that an adolescent's age did not always reflect their level of maturity, which they felt was more important in determining capacity for independent decisions.

“[P]eople don't mature the same way, you can find an adolescent who is 12 and is well developed in the brain but you find one who is 15 but he is still not very mature…”—[Participant 7, Public Health Official]

While age was still viewed as a relevant factor for assessing the decision‐making capacity of adolescents, participants suggested that other factors be considered, including mental health, cognitive development, reasoning, support network, and the physical and social environments in which adolescents were raised. For example, when asked what factors are useful to assess the capacity to consent in adolescents, one respondent said:

“Age of course. It's also good to look at the environment around this adolescent in terms of autonomy. By environment I mean, are they exposed to making decisions, being independent much earlier in their lives or not, that sort of family upbringing.”—[Participant 18, IRB/ERC Member]

### 3.2 Policies should evolve alongside changing societal views about adolescent autonomy

Participants identified missed opportunities to address adolescent HIV, which they partially attributed to the lack of response in HIV policies and interventions to changing patterns in sexual behaviours and risks among adolescents.

“We are being told 9‐year‐olds are having sex. They are not having sex with dolls; they are having sex with people who can transmit diseases. They should be allowed (to access HIV testing and care) to protect themselves against HIV… Is the policy in place to let us feel right and religious and moral, or is to protect and work for adolescents? I feel that the policies need to change completely; the laws need to change to reflect current society.”—[Participant 11, Lawyer]

When asked specifically about how current policies around consenting in HIV testing, care and research could change, almost all participants believed that the current default age threshold of 18 for making independent decisions should be adjusted. Only one participant thought that existing guidelines had enough flexibility. Others provided a wide range of alternative age cut‐offs, ranging from 11 to 24 years, with the most frequent suggestion being to lower the threshold to 15 or 16. Lowered ages reflected the belief that adolescents were maturing faster than earlier generations in Kenya, and were more familiar with the risk factors of HIV and care benefits given the spread of informational HIV campaigns and access to information via the Internet.

“[Y]ou know today, the world has become very digital and they learn quicker than before….” [Participant 7, Public Health Official]

One educational expert advocated for policy change specifically to reflect these changes in the world.

“[T]hese children are maturing early; what a child of 16 years then would think about then is not what they are thinking about now. Now they are very fast, a child who is hardly 7 years would tell you things that you don't imagine. So there is need to look at those policies or re‐look at the policies.”—[Participant 1, School Administrator/Teacher]

Some participants suggested that adjusting the age cut‐off alone was not sufficient to address missed opportunities in adolescent HIV. Participants advocated for campaigns to sensitize caregivers and the community to the current behavioural patterns of adolescent HIV and ethical conduct of research, as stigma and misunderstanding of these topics were considered major barriers to adolescent participation in HIV testing, care and research.

“First and foremost, we need to enlighten the parents and the society so that they know (the situation). We shouldn't bury our heads in the sand and assume that these children are not [sexually] active… When a child says they want it (PrEP), you only need to know what could have happened. And if that happened, you now know how to guide, so that it doesn't happen again.”—[Participant 1, School Administrator/Teacher]

Most participants believed that current policies regarding consent practices in HIV care and other sexual and reproductive health services should be periodically formed in response to evolving values, new technology and improved access to information. They suggested a participatory approach to reviewing policies and guidelines with the broad involvement of multiple stakeholders.

“[I]t has to be based on a lot of consultations so that nobody is left behind, nobody says, ‘no, they never seek for our opinion, they don't value our opinion, they don't like think we can provide this kind of information so include everybody on board’.”—[Participant 8, IRB/ERC Member]

While agreeing that adolescents were already frequently making independent decisions, some participants expressed concerns that adolescents might make suboptimal decisions due to a lack of experience or in response to coercive adult figures, peers or the internet.

“They might be swayed easily and in that, that might expose them easily to the predators, because their mind is not mature enough… maybe they see another person suffering from TV or from Cyber, whatever source, they might try [something], they might [make] whatever decision because they don't know what it takes to live safe….”—[Participant 7, Public Health Official]

Yet, many participants believed that adolescents should be given greater opportunities to make their own decisions, allowing them a deeper level of investment in the decision and the subsequent outcomes, and the chance to learn from active participation in the process.

“[I]t's necessary that the adolescents be given an opportunity to make decisions, because when an adolescent is given an opportunity to make a decision, then they own up [to] the process of decision‐making because they are part of it, but many times that is not what happens under most circumstances.”—[Participant 3, School Administrator/Teacher]

Participants viewed adults as key providers of empowerment and support for adolescents during this transitional time when adolescents’ decisional capacity was developing. Caregivers were commonly seen as the primary sources of support for adolescents, especially for younger adolescents (<15 years) and decisions related to health.

“It is about being with them, listening to their challenges, encouraging them and sharing with them the current trends in health issues: what is available, what works, and what doesn't work pretty well. You have to give them options and those must be guided options. Though not every guardian or parent is well informed in health issues, yet the role still lies with you. If you don't understand, there are people who understand, so you can link them (adolescents) to people who understand.”—[Participant 8, IRB/ ERC Member]

When primary caregivers were not available, participants felt that other key adults, including relatives, teachers and healthcare workers, trusted by adolescents could step in to provide support for decision‐making.

“It varies, it could be a healthcare worker it could be a parent, it could be a sibling…it should be someone that the adolescent is completely comfortable with and they relate well together and not someone who has coercive power over them. I think for me that would be the determining factor. Is it someone that you easily relate with, is it someone that you easily work with and they are able to get along?”—[Participant 14, Lawyer]

However, there was a lack of consensus on how alternative support figures might be engaged in practice, including how trusted adults should be identified, whether to engage them if caregivers are also available and whether trusted adults can have legal decision‐making authority. While legal guidance [41] indicates that any person acting with parental responsibility has that authority (i.e. an adult who has the duty to provide an adolescent with food, shelter, clothing, medical care and education, typically the mother and/or father), it is unclear how often this is followed in practice. There was consensus that building trusted relationships with adults that empowered adolescents by providing knowledge and options, involving them in decision‐making and respecting their preferences could improve their ability to make independent and responsible decisions. Within schools, administrators and teachers frequently referenced practices like class assemblies and access to peer counsellors as ways to empower and support adolescents.

“[W]e are trying to counsel them to help them gain ownership of what they are doing. What has been the issue here with these students is that they have been shaped to only live with rules that are given.”—[Participant 2, School Teacher/Administrator]

## 4 DISCUSSION

This qualitative study was designed to identify the views and insights of stakeholders with relevant experience regarding the policy for HIV testing, care and research participation among adolescents in Kenya. Policy stakeholders found legal approaches relying on age cut‐offs alone to be too restrictive. They advocated for more flexibility to assess adolescent capacity for independent decision‐making that incorporate maturity, risks and benefits, and context. Participants further endorsed a periodic review of laws and guidelines to account for changing societal norms and health behaviours. Finally, stakeholders described the important roles of adults in supporting adolescent decision‐making. As this study was conducted before the COVID‐19 pandemic, and gaps in HIV testing, care and research for adolescents have grown since that time, research on policies regarding adolescent inclusion is only of greater relevance [42].

Our results support and extend prior findings suggesting that engagement of adolescents in HIV testing, care and research is complex, requiring policy reform. Respondents expressed concerns over parental permission as a barrier to adolescents accessing health services and research. This aligns with previous studies showing that different types of relationships (e.g. with caregivers, partners or healthcare workers) play a critical role in adolescent use of HIV testing services in Kenya [32]. Moreover, Marsh and colleagues similarly found that community members believed adolescents’ increased access to information through the internet [43] enhanced their capacity for decision‐making about research. Furthermore, healthcare workers have expressed the desire to help minors seeking HIV testing who arrive at clinics without caregivers, even for those who cannot legally consent independently [32]. Our respondents felt that more flexibility was necessary for HIV testing and treatment, but that greater caution is required for research participation, particularly for higher‐risk research.

Notably, however, allowing independent adolescent consent can be controversial. For instance, adolescents sometimes desire greater autonomy than their caregivers or healthcare workers think appropriate [32]. Even for adults, community members have suggested that healthcare workers should not always assume capacity and more regularly assess it [44]. By contrast, some ethicists have argued for a default of allowing individuals to provide independent consent regardless of age [45]. The middle ground that our stakeholders endorsed—policies that allow healthcare workers greater flexibility, while maintaining default ages when people are deemed to have the capacity under the law—may prove to be more reasonable than either of these extremes.

In general, our findings suggest that providing a patchwork of exceptions to allow independent adolescent consent, as in Kenya and many other countries, is insufficient to address the needs and capacities of adolescents. A clearer, evidence‐based policy framework would be ideal. Furthermore, our findings suggest that there may be an appetite for reform to go beyond strict age cut‐offs. Participants viewed age as an imperfect proxy for decision‐making capacity. Some younger adolescents may have the capacity but be unable to access services if parental permission is required. By contrast, some older adolescents may have legal authority but need additional support to make good decisions.

One way to implement such reform would be to pass “mature minor” laws—laws in certain jurisdictions that allow clinicians to determine when adolescents are mature enough to make healthcare decisions. The greater flexibility allowed by these laws may offer advantages that age cut‐offs cannot, and their relative simplicity may make them easier to implement. For example, in South Africa, PrEP is permissible for persons over 12 years if clinicians determine they have the mental capacity to understand the benefits, risks and implications [46]. Adopting policies that move beyond age cut‐offs could allow clinicians to account for the range of capacity among adolescents, as well as evolving societal views about adolescents’ capabilities, which may more accurately reflect adolescents’ ability to make informed decisions [23, 47, 48].

Importantly, “mature minor” laws that allow clinicians greater discretion should be accompanied by training and guidance on how to make capacity determinations efficiently and minimize the potential for bias [49, 50]. Adapting existing tools to measure adolescent's decision‐making capacity, such as the Semi‐Structured Comprehension Interview [51, 52], to make them feasible in international contexts could help guide clinicians in making these assessments quickly and fairly. Additionally, while stakeholders desired greater flexibility for healthcare decisions, they were more cautious about research, particularly for riskier studies. Participants commonly preferred caregiver involvement in research consent processes involving adolescents, while acknowledging that caregivers could be a barrier to conducting research with adolescents. It has been well‐documented that requiring parental permission can compromise the scientific validity of research by resulting in low enrolment rates or selection bias [12, 53]. Given the potential value of extending “mature minor” laws from the clinical to the research context, other protections may help allay stakeholder concerns. For example, “mature minor” laws should probably not exempt studies from existing regulatory requirements for paediatric research. And as with waivers of parental permission, “mature minor” laws might only be applied to research that poses a minimal net risk.

Furthermore, our study highlights that merely considering *who* has the authority to make a decision may not be enough to support quality decision‐making. Our respondents—and particularly IRB members and policymakers—noted the importance of engaging caregivers who already provide support for adolescents on a daily basis to empower adolescents in decision‐making when possible and appropriate, as reported in other studies [54, 55]. Sometimes, individuals can be brought in to offer additional support in decision‐making, rather than to exercise veto power [56]. Caregivers could, therefore, be part of the consent process as a source of support for adolescents to make informed decisions and cope with challenges, without having the authority to make decisions *for* adolescents. Even for minors who are deemed to be “mature,” such an approach could demonstrate respect for adolescent autonomy and authority, while also allowing caregivers to provide critical support [23].

When caregiver support is not available, respondents broadly considered ways adolescents could receive support from other trusted figures within the community [32]. More research on alternatives to parental or caregiver involvement is needed. Clearer legal avenues to engaging trusted adults who are not caregivers may be beneficial in some cases. Working with IRBs may be necessary to identify situations in which trusted adults can guide adolescents. Alternatively, encouraging adolescents to engage with other adults they trust could become a recommended, but not required, part of the process for studies where independent adolescent consent is permitted.

Additionally, some researchers have sought community approval as an additional safeguard for adolescent participation in research when parental permission is waived [57, 58], particularly for adolescents whose parents cannot be engaged, such as homeless children and those who have been orphaned. In South Africa, conditions for waiving parental permission include: justifying the need to waive parental permission to the Ethics Review Committee, ensuring the risk is minimal and participants are over 16 years of age, and obtaining prior approval from the host community [59, 60]. While these examples of safeguards may be instructive for research oversight committees and policymakers about ethical ways to permit greater flexibility, more work is needed. In particular, guidance regarding what types of community engagement can effectively function as safeguards is needed, along with efforts to ensure the voices of marginalized groups within communities are heard [58].

Finally, our results further highlight the need to regularly revisit laws, policies and recommendations to ensure they do not become ossified and reflect evolving societal views. Most participants felt that adolescents in Kenya now mature faster than previous generations given the broad dissemination of information through the internet and social media. While some data suggest that adolescent girls are empowered by greater access to health information through the internet, social media exposure can harm mental health, and more research is needed to better understand these effects [61, 62]. Policies around consent practices in HIV testing, care and research could be reviewed periodically with contribution from all stakeholders to address changes in culture and technology between generations. In the United States, commentators have argued that a process is needed for regularly revising the U.S. Common Rule to reflect societal changes [63], as already exists for research regulations in Japan [63, 64]. Our respondents further expressed that a participatory approach to revising national policies and guidelines in a way that engages relevant stakeholders would be ideal.

Our study has limitations. We recruited a relatively small sample of 18 stakeholders to represent policy‐adjacent decision‐makers in Kenya; their views do not necessarily represent the perspectives of all relevant stakeholder groups, including adolescents (whose data will be published separately). We also interviewed only three school administrators; future studies focusing on how policies function in school settings would be valuable. Two audio recordings were corrupted, so we were unable to use full transcripts or quotations from those interviewees but relied on detailed notes from the interviewers instead. The length of each interview also varied considerably, ranging from 16 to 67 minutes. As some participants had limited time to contribute, this may mean that some stakeholders had more substantial contributions to our results than others.

## 5 CONCLUSIONS

As adolescents remain a significant proportion of people living with HIV, policy flexibility may be an important tool to help end the global HIV epidemic. Our results suggest that policy stakeholders support legal reform to move beyond strict age cut‐offs for consent to HIV testing, care and research. Working with parents and other trusted adults when appropriate and maintaining risk/benefit protections for paediatric research even when adolescents can consent independently can help to protect adolescents’ rights and avoid exploitation. Building a regular process of revision into national policies and guidelines around HIV testing, care and research involving adolescents may ensure that policies remain responsive as societies evolve. Future research should examine how best to support decision‐making for adolescents who lack parental support and to conduct a systematized and routine process of policy reform for adolescent engagement that is inclusive of relevant stakeholders.

## COMPETING INTERESTS

The authors have no competing interests to report.

## AUTHORS’ CONTRIBUTIONS

GJS, PK and KA obtained study funding and served as study principal investigators. KBS led protocol development and study oversight for the qualitative work with assistance from KW, SKS, JD, JB and JN. JB recruited and enrolled participants. WOA and HM conducted the interviews and provided feedback during the analysis. HL led the ELSI stakeholder analysis with assistance from EH. HL, EH, KBS and SKS developed the codebook, coded and analysed the study findings. HL, KBS and SKS wrote the initial manuscript. All authors critically read and revised the final manuscript.

## FUNDING

This research was sponsored by the National Institute of Child Health & Human Development (UG3 HD096906) and Prevention and Treatment through a Comprehensive Care Continuum for HIV‐affected Adolescents in Resource‐Constrained Settings (PATC^3^H). Additional support was provided by the UW Global Center for Integrated Health of Women, Adolescents and Children (Global WACh) and the University of Washington CFAR (P30 AI027757).

## Supporting information


**Supplementary Material 1**. ELSI Stakeholder Semi‐structured Interview Guide.

## Data Availability

Data cannot be shared publicly because of the sensitive nature of qualitative data. The data are available from the University of Washington for researchers who meet the criteria for access to qualitative data.
