# Supplementary material for: “[T]he laws need to change to reflect current society”: Insights from stakeholders involved in development, review or implementation of policies about adolescent consent for HIV testing, care and research in Kenya
Source: J Int AIDS Soc. 2023 Jan 15;26(1):e26057. doi: 10.1002/jia2.26057 (PMC9841068; doi:10.1002/jia2.26057)
Supplement: Supplementary file 1 — Supplementary Material 1. ELSI Stakeholder Semi‐structured Interview Guide. [file JIA2-26-e26057-s001.docx]

***Supplementary Material 1. ELSI Stakeholder Semi-structured Interview Guide***

**Interviewer Instructions:** Copies of the informed consent form should be provided to the participant and read aloud for the benefit of those who cannot read. Participants should be provided an opportunity to ask any questions. Verbal agreement to participate should be taped.

The following set of questions is a guide. Try to ask all the questions below in the order given, but it is more important to maintain the flow of discussion. Suggested probes have been included.

**Welcome and Introductions**

**Before turning on the recorder, start with the following introductory script:**

*Hi, my name is ____________. Thank you for agreeing to talk to me today. As mentioned earlier, I am a researcher and I am trying to learn more about current practices for providing services for HIV-infected adolescents. I am especially interested in how to balance adolescent’s developing autonomy with providing appropriate support when adolescents are making medical and research decisions. We hope that the information you tell us can help improve the process of getting medical care for adolescents and young people with HIV.*

*This project is funded by the US National Institutes of Health.*

*Thank you for allowing us to spend time with you today.*

*Before we begin talking with you about your experiences, we want to learn a little bit more about who you are by asking a few short questions.*

**Ask and complete demographic survey now.**

*Thank you. We are now ready to begin our longer discussion. During this conversation, I am interested in understanding all of your thoughts, experiences and opinions. I will ask you questions that you are free to answer in any way you wish. Your opinion is very important to us. You do not have to answer any question that you are not comfortable with. If you want to stop the interview at any time, just tell me and we can stop.*

*There is no right or wrong answer to anything that I ask. If a question is unclear to you, please feel free to ask me to explain it.*

*The research team may take short sections of what you say and share them with non-study members. The research team will take information about your age, education, and experience and combine this information with others participating in these interviews.*

*I would like to record the interview so I don’t miss anything that you say. I will not include your full name in the recording. Your name will not be on any study documents. Your answers will be kept confidential.*

*Is it okay if I record our discussion? [Wait for the participant to give verbal consent to recording; those who decline at this stage will be asked to leave the group and not participate]*

*I am turning on the recorder now.*

**Before beginning interview questions, please read the following script for the recording:**

*Today is [day of week], [month, day, year] and it is now [time of day]. I am speaking with participant [participant ID].*

*Can you confirm for me again, by stating yes or no, that you are willing to participate in the conversation today? Also, can you confirm if you agree to be audio-recorded?*

**You are now ready to begin asking the questions outlined below.**

**Semi-Structured Interview Questions**

First, I would like to learn more about your involvement with adolescents. Please tell me a little bit about your professional role and how it relates to adolescents with HIV.

Can you tell me more about how your professional role involves thinking about adolescent decision-making?

- In what types of situations do you think about adolescents being involved in the decision-making process?
- In what situations do you think about adolescents being able to give consent for themselves to participate?
- In your professional role, what policies govern adolescent decision-making abilities?

Who are the best people to help support adolescent decisions about medical care?

- What is the role of a parent or guardian in helping adolescents make decisions?
- Who else might help support adolescents?
  - Under what circumstances can these supportive people step in if parents aren’t available?
  - Is there a role for peers in supporting young people with their decisions? What might that role look like? Why?
  - Is there a role for other adults (not a caregiver or guardian) in supporting young people with their decisions? What might that role look like? Why?

Do you think adolescents should be able to make independent decisions to get an HIV test? Why?

- Under what circumstances is this acceptable? [For example: Does it depend on age, gender, maturity, education, behavior, fear of parental involvement, region of the country?]
- In what circumstances is this unacceptable?

Do you think adolescents should be able to make independent decisions to get HIV care? Why?

- Under what circumstances is this acceptable? [For example: Does it depend on age, gender, maturity, education, behavior, fear of parental involvement, region of the country?]
- In what circumstances is this unacceptable?

Do you think adolescents should be able to make independent decisions to get HIV prevention (PrEP)? Why?

- Under what circumstances is this acceptable? [For example: Does it depend on age, gender, maturity, education, behavior, fear of parental involvement, region of the country?]
- In what circumstances is this unacceptable?

Do you think adolescents should be able to make independent decisions to enroll in research studies? Why?

- Under what circumstances is this acceptable? [For example: Does it depend on age, gender, maturity, education, behavior, fear of parental involvement, region of the country?]
- Does the type of research activity matter?
  - What about participating in surveys?
  - What about participating in interventional studies?
- In what circumstances is this unacceptable?

What types of factors to you consider most frequently when thinking about adolescent autonomy and decision-making? Why?

Do you think that if an adolescent was determined to be mature and able to understand the research, it is ok for them to participate without their parents or anyone else helping them make decisions?

Do you think that if an adolescent was assessed and supported by a trusted adult(not parent or caregiver) in making a decision (e.g., by helping the adolescent think about longer term consequences, or resist peer pressure), that they could make a decision without parental support?

In your field, what are the best ways to change policies about when adolescents are considered able to make autonomous decisions?

Do you think current policies should change? Why?

Today, we talked about adolescent autonomy and who helps adolescents make different types of decisions about their HIV care, testing for HIV, HIV prevention and involvement in research studies.

Before we end, is there anything else you would like to add?

Do you have any questions for me before we end our conversation today?

Thank you very much for your time and for all the helpful information you have provided!
